# A Micellar On-Pathway Intermediate Step Explains the Kinetics of Prion Amyloid Formation

**DOI:** 10.1371/journal.pcbi.1003735

**Published:** 2014-08-07

**Authors:** Erwan Hingant, Pascaline Fontes, Maria Teresa Alvarez-Martinez, Jacques-Damien Arnaud, Jean-Pierre Liautard, Laurent Pujo-Menjouet

**Affiliations:** 1 CI^2^MA, Universidad de Concepción, Concepción, Chile; 2 INSERM U710, Université Montpellier 2, Place E. Bataillon, 3eme étage, Montpellier, France; 3 Etablissement Confiné d’Expérimentation, Plateforme RAM, UMS 3426-BioCampus, Place E. Bataillon, UM2, Montpellier, France; 4 Centre de Recherche sur les Pathogènes et Biologie pour la Santé, CPBS UMR5236, Université Montpellier 2, Place E. Bataillon, Montpellier, France; 5 Université de Lyon, CNRS, Université Lyon 1, Institut Camille Jordan UMR5208, Villeurbanne, France; 6 INRIA Team Dracula, Inria Center Grenoble Rhône-Alpes, France; Accelrys, United States of America

## Abstract

In a previous work by Alvarez-Martinez *et al.* (2011), the authors pointed out some fallacies in the mainstream interpretation of the prion amyloid formation. It appeared necessary to propose an original hypothesis able to reconcile the *in vitro* data with the predictions of a mathematical model describing the problem. Here, a model is developed accordingly with the hypothesis that an intermediate on-pathway leads to the conformation of the prion protein into an amyloid competent isoform thanks to a structure, called micelles, formed from hydrodynamic interaction. The authors also compare data to the prediction of their model and propose a new hypothesis for the formation of infectious prion amyloids.

## Author Summary

Understanding the mechanism of prions is an important issue. Indeed, it involves a mechanism modifying the structure of the proteins that are of high interest in theoretical biology. Knowing the underlying mechanism that leads to prion disease could help further investigations in the world of amyloid disease and for example the so-called Alzheimer's disease. The theory of prion, also known as Protein-Only, has been widely studied. Nevertheless no mathematical models are able to reproduce the phenomena *in silico*. This suggests a lack of information in the theory. Here we propose a new model, built with a new approach theory that fits experimental data in a very satisfactory way. This model, together with experiments, maintains the idea that an intermediate conformation of the protein helps the disease to spread. Besides, this work is an excellent example of a strong interaction between mathematical modelling and biological approach. Indeed, because of a strong discrepancy between theoretical results of the early original model and biological data on pathological prion formation, the team of biologists decided to investigate more closely their experiments. They came out with a new discovery: the crucial role of micelles in the pathological conformation of the prion protein.

## Introduction

Transmissible spongiform encephalopathies, or prion diseases, are a group of fatal neurodegenerative disorders of humans and animals. The pathogenic process is typically associated with conformational conversion of a cellular protein, called prion or PrP^C^, to a misfolded isoform, called PrP^Sc^. The “protein-only” model asserts that this rogue PrP^Sc^ represents the infectious prion agent, self-propagating by binding PrP^C^ and inducing its conversion to the abnormal PrP^Sc^ [1], [2]. This scenario was quantitatively described as a nucleation-dependent amyloid polymerization [3], [4]. It is now generally accepted that the prion development process results from an amyloid polymerization after an initial nucleus formation in the very early phase of protein aggregation. Models based on nucleation-dependent polymerization [5]–[7] describe a molecular mechanism at the origin of the formation of large protein aggregates, by involving thermodynamically unfavourable steps that become favourable when the nucleation kinetic barrier is reached. As a striking consequence of these models, the unfavourable first steps can be bypassed by seeding with preformed polymers. However, due to the transient nature of the initial nucleus, our understanding of the interactions that form this initial structure is very sparse and thus the understanding of species allowing the prion proteins to overcome the strong kinetic barrier to form a specific amyloid conformation is highly limited [8]. This may have led to one of the most notable persisting fallacy claiming that the Lag phase of prion proliferation, defined as the required phase for the nucleus formation [3], [9], reflects the unfavourable nucleation phase. This idea was challenged by experimental results obtained by numerous authors who revealed a linear dependence of the lag time (denoted by 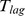) to monomer concentration not exceeding a nucleus size of about 2 monomers [10], [11]. This result, also found for some other spontaneous amyloid-forming proteins [12], was generally attributed to an accumulation of large off-pathway species whose formation is competitive with the on-pathway processes that lead to amyloid [11], [13]. However, in the case of hamster rPrP (Recombinant Syrian Hamster Prion Protein 90–231) polymerized *in vitro*, we previously found no kinetic evidence for an off-pathway [10]. Consequently, we propose that an additional on-pathway step is necessary to explain the results observed. We hypothesize that this new stage stands very likely for a first step. It would occur before nucleation, because experimentally, we were able to show that seeded polymerization always begins after a time delay that can be interpreted as the time needed to generate active monomers [10]. We show indeed that micelles are formed, which leads to an amyloid competent isoform of the prion protein (denoted by PrP*) considered as a necessary step to induce nucleation and amyloid polymerization. This hypothesis is supported by [14], [15] where it is found that lipid interactions play a key role in the conformation of PrP^C^ into PrP^Sc^ by β-sheet enrichment, see Sec. Discussion. We assume here that a similar interaction occurs in our experimental condition by pure PrP^C^ interaction with the help of micelles formation. To analyse the consequences of this hypothesis, we develop a quantitative model with an explicit description of the microscopic processes, and we compare experimental data with the results predicted by the model.

## Results

### Micelles characterization

**Figure 1:**
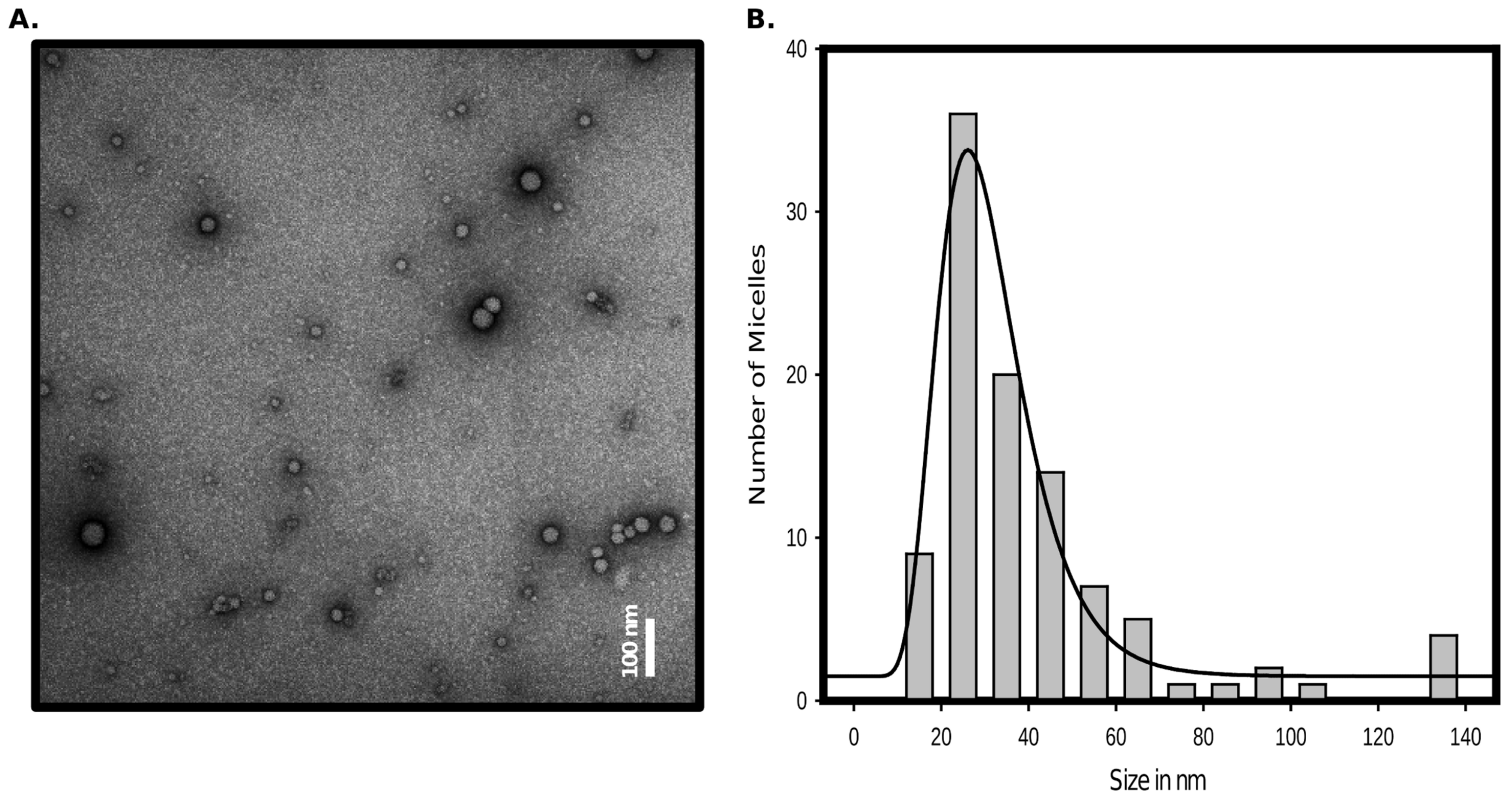
Micelle-like structures. A. Electron microscopy view of the rPrP^C^ few minutes after solubilisation (see Materials and Methods). Samples were adsorbed on carbon/formvar film and negatively stained by 2% uranyl-acetate, and examined on a Jeol 1200 EX. B. Diameter measurement of the micelles observed on one sample as described in item A.

In order to find out what types of structures could be involved as an on-pathway, we have performed a time dependent electron microscopic analysis during polymerization of hamster rPrP. Few minutes after dilution into polymerization buffer, we observed spherical structures looking like rigid micelles (Fig. 1A and Fig. S6 in Supporting Information). The size distribution fits well with a log normal distribution (Fig. 1B). The size of these spheres was heterogeneous with a mean around 30 nm. A sphere of 30 nm diameter reaches a surface area of around 30 nm^2^, the rPrP have a diameter (thanks to Protein Workshop v1.0 and PDB ID 1B10) of about 1.5 to 2 nm thus the sphere contains about 1000 proteins on its surface. In order to ensure that these structures are formed of rPrP, we decided to label them with antibodies. This method clearly identified spheres consisting of rPrP (details in Fig. S1).

### Micelles quantity correlates with fibrils in a precursor relationship

**Figure 2:**
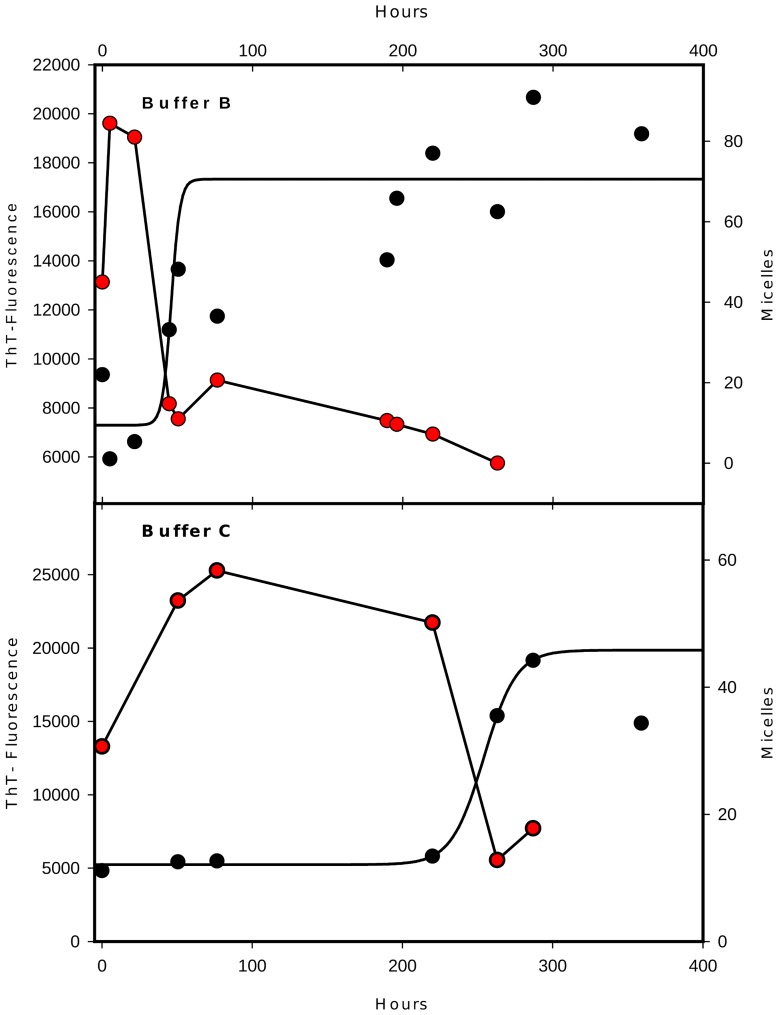
Micelle-like structures precede amyloid fibril formation. Comparison of the dynamics of micelles and amyloids. Amyloid formation was measured by fluorescence of ThT (in black circle) and micelles number (in red circle) counted directly on arbitrarily selected grids observed at the same magnification (top in buffer B and down in buffer C).

In a previous study [10], we showed that polymerization kinetics could not be explained by the existence of an off-pathway. Thus an important question remains: what exactly is the role of micelles in the polymerization mechanism? To answer this, we decided to analyse the evolution of the micelle quantity during polymerization kinetics. Qualitative analysis using electron microscopy revealed that the number of micelles is important a few minutes after dilution into polymerization buffer, and then rapidly decreases when fibrils are formed (Fig. 2). A semi-quantitative analysis of the amount of round shape structures suggested a precursor relationship between the micelles and fibrils (Fig. 2). This was established in two different buffers exhibiting very different lag phase and thus showing that it is very likely a common feature of *in vitro* prion polymerization.

### Quantitative model of polymerization including on-pathway micelles

To quantitatively analyse the consequences of an on-pathway micelle intermediate, we built an *a priori* model describing the different steps with the microscopic processes involved and their contributions to the whole system. From this microscopic model it is then possible to quantify macroscopic data, such as micelles, polymers and monomers concentration. The model can be detailed into four main parts.

- **Phase 1:** Formation of dependent PrP micelles through a growth phase with addition and loss of monomers as a cluster dynamics. It corresponds to the early phase in Fig. 2 where micelle quantity increases (curve with red circles) while the PrP^Sc^ still remains constant (curve with black circles).
- **Phase 2:** Micelles help the transition towards a new structure (PrP*) that is stabilized in the micelle itself and is released as an isoform monomer. This is the main hypothesis of our work. Production of PrP* occurs all along the process until the stabilization of PrP^Sc^ is reached.
- **Phase 3:** The free isoform (PrP*) is able to reach the nucleation barrier and then nucleates. It corresponds to the phase in Fig. 2 where micelles (red circles) start to decrease while the quantity of polymers (black circles) remains constant.
- **Phase 4:** Nucleation promotes polymerization of PrP^Sc^ and fibrils split when size increases, leading to a rapid polymer (black circles) growth phase. Here, it corresponds to the fast growth phase of PrP^Sc^ in Fig. 2.

### Microscopic description of the model and kinetic equations

A standard approach to formalize particle interactions between monomers, micelles and polymers, is to describe the transition rates between the states of the system through kinetic schemes. Well-known in chemistry, such a modelling is also used for polymerization models [4] and according to this method, it is then possible to write the differential equations of the concentration describing the dynamics of each quantity involved in the system.

In order to give the clearest insight as possible of the model elaboration process both for biologists and mathematicians, let us consider the 4 phases listed in the last section and progressively introduce the microscopic processes involved together with their kinetic schemes and the corresponding equations.

#### Phase 1: Formation of dependent PrP micelles

**Figure 3:**
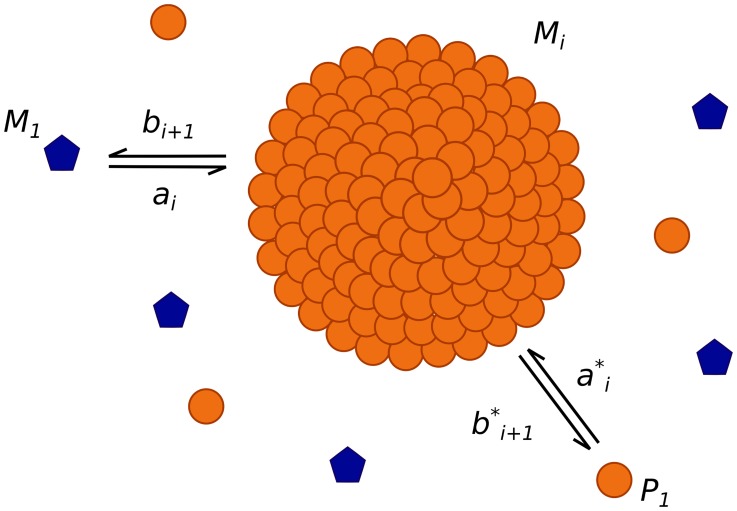
Schematic representation of a micelle and interactions with monomers. The blue pentagons represent the PrP^C^ monomers, the big orange spherical structure stands for a micelle consisting of 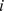 monomers, and the orange spheres (out of micelle) are the PrP* monomers stabilized by micelles. Both types of monomers, PrP^C^ and PrP*, interact with the micelles by association-dissociation with rates given by 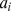, 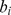, 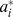 and 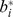 depending on the number 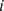 of monomers that form the micelle.

We denote by 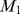 the PrP^C^ monomer population (blue pentagons in Fig. 3) and by 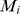 the micelles consisting of 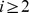 monomers (orange spherical aggregate in Fig. 3). We assume that micelles grow by addition of PrP^C^ one after the other, *i.e.* a PrP^C^ monomer will aggregate to a micelle consisting of *i* monomers to form a larger one consisting of 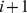 monomers. The rate at which this reaction occurs is given by a non-negative number 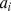 that depends on the size 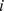 of micelles. We also assume that this reaction is reversible and a micelle of size 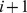 can release a PrP^C^ monomer to form a micelle of size 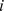. The rate of this reaction is given by the non-negative number 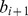, also depending on the size. Both reactions are represented in Fig. 3 by the interaction between the blue pentagon and the spherical aggregate. The kinetic scheme of these two reactions is given by 
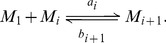
(1)


The differential equation describing the quantity change rates are written in function of the concentration of each particle type. We introduce then 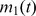 and 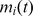 the concentrations, respectively of, monomers 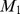 and micelles 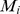 for 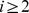 at time 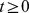. The flux associated to reaction (1), also called net rate, is then given by 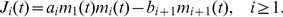
(2)


#### Phase 2: Transition to PrP*

We assume that the reaction, between PrP* monomers (orange spheres in Fig. 3) denoted by 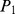 and micelles 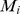 for 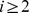, follows the same dynamics as the PrP^C^ monomers. Thus, the kinetic equation writes, for 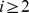, 
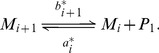
(3)


The rates 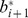 and 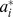 play the same role as in (1). They respectively stand for, the rate at which a micelle 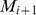 releases a PrP* monomer after having stabilized it and the rate at which a micelle 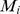 associates a PrP* monomers. If we denote by 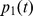 the concentration of 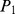 at time 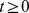, we obtain the net rate for reaction (3), 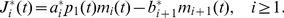
(4)


To summarize, reactions (1)–(3) can be combined and written as 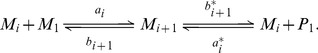
(5)


This shows how micelles help the transition from PrPC monomers to PrP*. Both depletion rates 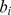 and 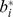 represent, in some sense, the probability that a monomer released by a micelle of size 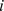 is stable in one of the two free isoforms, PrP^C^ or PrP*. The way we choose these coefficients in the model will strongly depend on the structure of the micelles: we assume here a 3D spherical micelle structure in agreement with *in vitro* observation, see Sec. General assumptions and Fig. 1A.

#### Phase 3: Nucleation

In this phase, we denote by 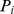 a small aggregate consisting of 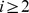 PrP* monomers up to a size 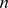. Here, 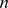 corresponds to the size required to form a so-called nucleus. We name these intermediate aggregates oligomers. The reactions involved in the formation of a nucleus 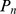 are the 
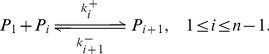
(6)


Coefficient 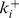 is the rate at which a small aggregate 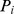 will bind a PrP* monomer to form an aggregate 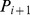. Here again, the reaction is reversible and 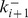 is the rate of the reverse reaction. The net rate associated to reaction (6) is written as follows: 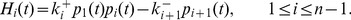
(7)


**Figure 4:**
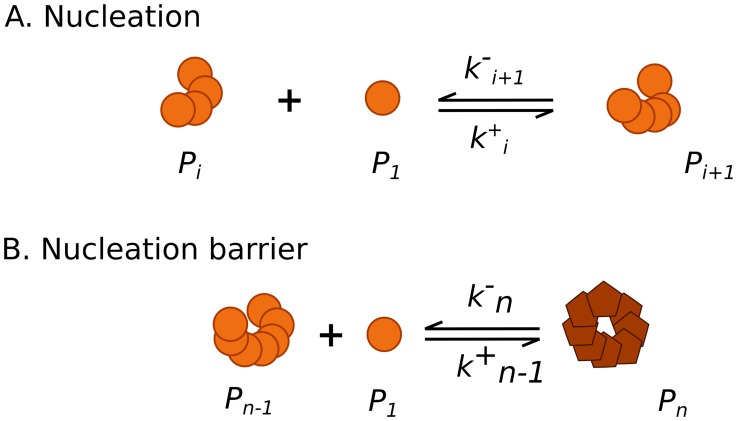
Schematic representation of the nucleation. A. The PrP* monomers form small aggregates called oligomers. B. When the oligomer reach a size 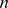, the oligomer structure becomes stable and forms a PrP^Sc^ polymer.

This process is called the nucleation because small aggregates are still assumed to be composed of PrP* (Fig. 4A). And these oligomers are unstable because the reaction is energetically unfavourable, namely 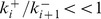. Thus, oligomers can easily release PrP* until their reach the critical nucleus size 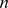. Once this size is reach, a so-called the nucleation barrier is crossed, *i.e.* an aggregate of size 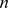 is formed, and monomers become attached to the newly created PrPSc isoform polymers (Fig 4B). An important hypothesis here, is that the PrP^C^ cannot reach this barrier which would be energetically to high while PrP* isoform would decrease considerably this level of energy necessary to reach the nucleus. This is the reason why the micelle role of converting PrP^C^ into PrP* is crucial here.

#### Phase 4: Polymers growth phase

**Figure 5:**
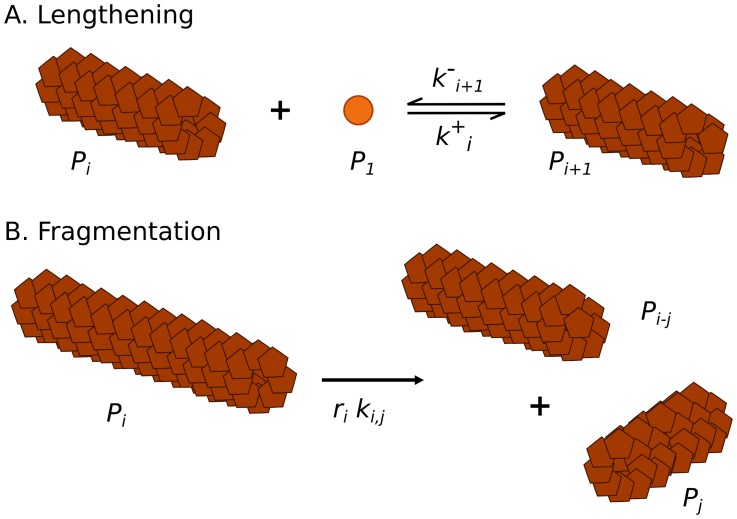
Schematic representation of the polymers dynamics. A. The PrP^Sc^ polymers 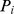 (consisting of 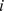 monomers) bind (or loose) PrP* at its ends. It is the polymerization (or de-polymerization process). B. Polymers split into two smaller pieces. A polymer 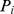 gives rise to two new polymers 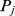 and 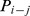 at a rate 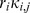.

After PrP* monomers have formed a nucleus of size 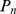, that is the minimal stable structure of PrP^Sc^, it polymerizes to form what we call polymers of PrP^Sc^ and denoted by 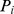 when it consists in 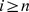 PrP* monomers (Fig. 5A). In this model, we assume that polymers only polymerize with PrP* monomers and not with the PrP^C^. Indeed, we assume that energy necessary to pass from PrP^C^ to the PrP^Sc^ isoform is much more important than the one to pass from the PrP* (stabilized by micelles) to the PrP^Sc^. Thus, similarly to the nucleation steps, we define 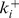 and 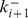, now for 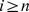, respectively the polymerization rates and depolymerization rates such that 
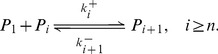
(8)


This step is thermodynamically favourable and 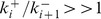. The net rate of reaction (8) reads as (7), *i.e.*

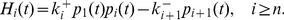
(9)


Finally, we assume that under our experimental conditions (agitation) both oligomers and polymers are subjected to a binary fragmentation, namely they can split into two smaller polymers (Fig. 5B). This phenomenon contributes to the growth phase by multiplying the possible polymerization sites and explains why the mass of polymers (Fig. 2) seems to increase exponentially after the lag time. Thus, we define a splitting rate, given by 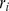, of a polymer 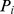 and the associate distribution kernel 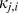. This latter gives the probability that a polymer consisting of 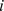 monomers, when it splits, gives rise to two polymers: one consisting of 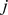 monomers and another of 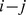. The reaction is 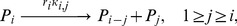
(10)


and the net rate is 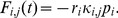
(11)


All these considerations give us the material to write evolution equations introduced in next section. These equations are the ones used for simulation in the next sections.

#### Derivation of the model

The kinetic schemes and fluxes derived in the previous section are summarized in Table 1. Using the reaction fluxes notations, it is then now possible to derive differential equations describing the dynamics of monomer, micelles and polymer formation at time 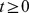 through their concentrations 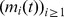 and 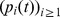. We recall that 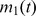 is the concentration of PrP^C^ monomer, 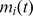 with 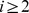 the concentration of micelles consisting of 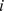 monomers, 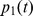 the concentration of PrP* monomers, 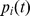 with 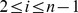 the concentration of PrP* oligomers, 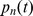 the concentration of nucleus and 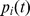 with 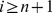 the concentration of PrP^Sc^ polymers consisting of 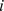 monomers.

**Table 1:** Reaction scheme and their related fluxes.

Description	Reaction scheme	Reaction fluxes
Micelle formation and PrP* released	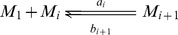	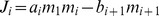
	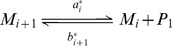	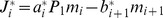
Amyloid polymerization	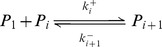	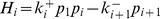
Amyloid splitting	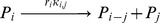	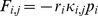

We denote 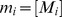 and 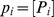, the respective concentrations.

We assume that the coefficient rates 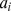, 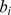, 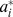, 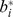, 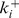, 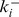 and 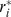 are non negative. Moreover, for any 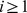 the kernel 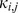 is non-negative such that for 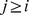, 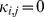. This latter equality means that a polymer 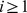 cannot split into bigger polymers, but only in two smaller pieces. We assume that 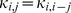 for any 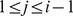. This symmetry assumptions means that we cannot distinguish between the fragmentation of a polymer 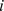 into two polymers 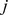 and 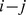 or two other 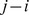 and 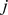, it is the same reaction. Finally, it is a probability kernel, for any 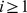, that is
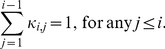
(12)


We are able now to write the differential equations associated to each concentration. First, the PrP^C^ monomers is involved in all the reactions (1) and its dynamics is given by summing all the flux set up in (2): 
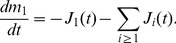
(13)


The flux 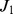 appears twice because there is two monomers involved in the first reaction. Then, the evolution of micelles concentration is given by 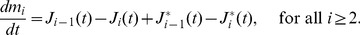
(14)


Indeed, there are four reactions that affects a micelle 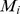. Two are of type (1) with the PrP^C^ monomers associated to the net rates 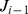 and 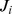 while the two others are of type (3) with the PrP* monomer associated to the net rates 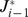 and 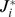. Reactions (3), (6), (8) and (10) involve PrP* monomers. The dynamics of 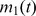 is written as follows: 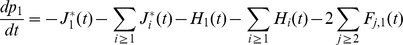



In particular, if 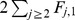 accounts for all fragmentations such that a polymer 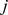 leads to a polymer of size 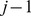 and a monomer. The reactions are counted twice because of the symmetry of the reaction. Finally, oligomers and polymers follows the same dynamics, given by reactions (6), (8) and (10) with their associated fluxes. It is written as follows: 
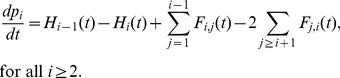
(15)


The fragmentation terms account for all the splitting of a polymer 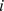 to give two smaller ones of size less than 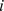 and the creation of a new polymer 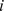 from the fragmentation of a bigger one.

In conclusion, the model we developed can be summarized as as a set of equations (13) to (15). The system has to be completed with initial conditions that depend on the experimental conditions, that is:
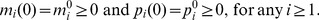
(16)


Before we analyse the system, we would like to emphasize the fact that this model describes the concentration of all micelles and polymers according to their size (number of monomers). Nevertheless it is possible to compute macroscopic quantity such as, 
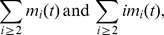
respectively the concentration of micelles and the *mass* of micelles which is the concentration of monomers in all the micelles. Similarly, 
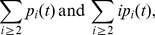
stands for the concentration of polymers and the *mass of polymers* that is the number of monomers in all the polymers. Finally, as we consider a closed system, with neither degradation nor production of monomers, it is then expected that the model preserves the total mass of monomers which is written as follows: 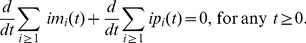
(17)


Indeed, summing all the equations of the system, and particularly thanks to the properties of the probability kernel 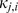, this conservation holds.

### Analysis of the experimental results based on this model

**Figure 6:**
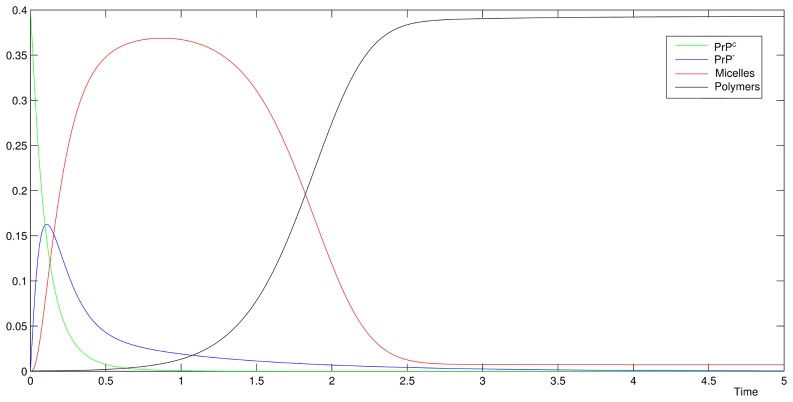
Qualitative behaviour of the model. Evolution normalized of the quantity: PrP^C^, PrP*, the *mass* of micelles and the *mass* of polymers. The dynamics are consistent with the experimental data shown in Fig. 2.

**Figure 7:**
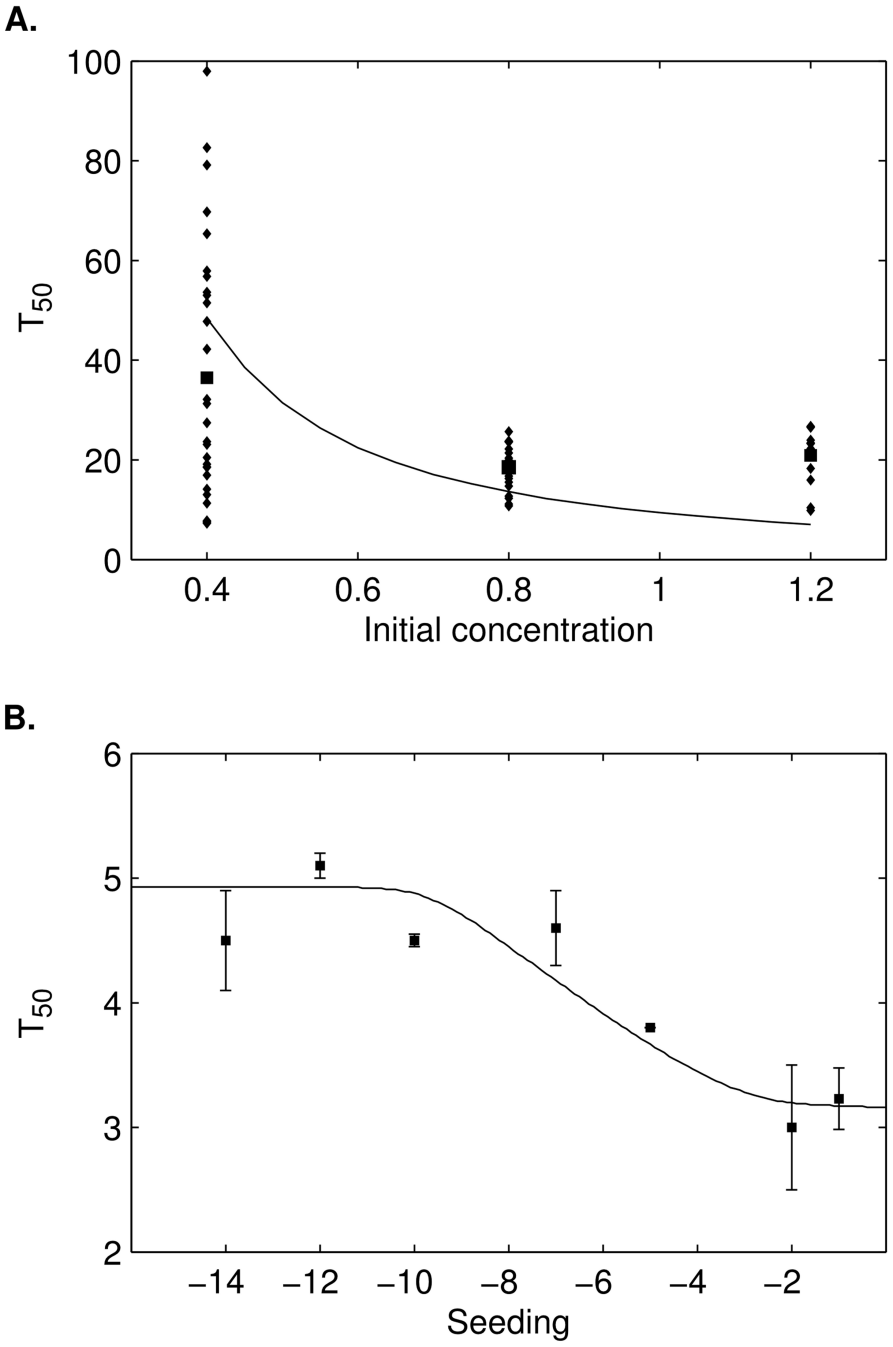
Half-time correlation between the model and experimental results. A. Diamonds are the 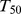 (in hours) obtain by fitting experimental data with a sigmoid (see [10]) for initial concentration of PrP^C^ equal to 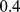, 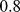 and 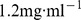. Squares are the mean value of the 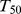 while continuous line is the value obtain by the model for a concentration of PrP^C^ varying from 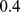 to 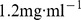. B. Squares stand for the mean 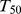 (in hours) with standard error of the mean *vs* the seeding concentration of PrP^Sc^ (in log-scale), obtained for a series of experiments. In each experiment the concentration of PrP^C^ was fixed to 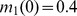 mg/ml while the seeding concentration of PrP^Sc^ given by 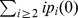 (mass of polymers) ranges from 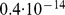 to 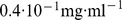. The result here gives the 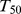 *vs* 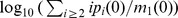. The continuous line corresponds to the simulations obtained with the model.

A qualitative analysis of the dynamics of micelles and polymers given by our model (see Fig. 6) is consistent with the one observed in experiments (see Fig. 2). As expected, the correlation between polymers formation and the decreasing of micelles concentration is connected with the PrP* formation. For this purpose, we assume in our simulations that PrP* monomers originate from micelles and do not exist before, *i.e.* their initial concentration is null. Furthermore, this model was built to analyse the lag phase and be compared to data. Several definitions of the lag time exist but they are mostly related to the half-time (denoted by 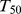), which is the time when half of the final polymerized mass is reached. One of the definitions links the 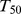 to the lag time by the relation: 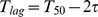, with 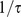 the maximal slope of the sigmoid [6] describing polymer dynamics with respect to time. This formula makes sense for a genuine sigmoidal equation, but here we cannot have any explicit solution to the model. Since the half-time 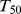 is better defined and more tractable on our data, we choose to use it to analyse our results. It is the possible to focus ourselves on four main results provided by the model:

1. We perform an analysis of the delay before polymerization starts when experiments are seeded with preformed PrP^Sc^ polymers at different concentrations. Note that nucleation steps are bypassed in this case since PrP^Sc^ already exists at the beginning of our experiment. Previous works [4], [16] predicted the disappearance of the lag phase (Fig. S2) and expected the half-time to go to zero. In Fig. 7B, we can see that these phenomena are absolutely not observed experimentally while, on the other hand, the model we introduced confirm perfectly these observations and fit the data correctly. Moreover, as expected the half time computed in our model does not converge to zero when the seeding concentration of PrP^Sc^ polymers increases.
2. Results in Fig. 7A show that the lag phase persists with an increasing concentration of PrP^C^ monomers considering the nucleation case (without seeding). That fact is consistent with the generation of active monomers in the system. The shape of dependency to the initial condition is preserved, *i.e.* a linear log-log shape, but it remains independent of the nucleus size (Fig. S5). It comforts the idea that the Lag time does not depend on a nucleation barrier. We emphasize that, for low concentration of PrP^C^, the dispersion becomes higher, this phenomenon could be explained by the stochasticity involved in the system and by-passed at high concentration when the interactions between proteins are facilitated. This has been studied in [17].
3. In the conventional model of nucleation, if the seeding is postponed, an increase of the 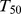 equal to the delay time before seeding should be observed (at least in the very first hours when the experiment takes place far enough from the 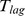). However, this dependency was not observed experimentally and the 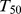 is less than the one expected. This suggests that a phenomenon occurs during the early phase without seeding, which accelerates polymerization when nucleus is introduced. Our model explains this phenomenon by formation of the amyloid competent isoform and results are shown in Fig. S4.
4. In the next step, we analyse the shape of the maximal speed distribution of polymerization (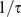) as a function of initial concentration. Our simulations appear to be qualitatively consistent with the experimental data, with concave shape of dependency (Fig. S3).

Taken together, these four points, allow us to conclude that experimental data corroborate our model. Furthermore, it suggests a simple explanation for the weak dependency of the lag time with initial concentration and it proposes a new interpretation of the overcoming kinetic barrier of the prion protein. *A posteriori*, the microscopic processes involving prion proliferation, built here, describe the observed *in vitro* macroscopic facts.

## Discussion

Evidences for the existence of micelle as an on-pathway during the formation of amyloid *in vitro* leads then to the question of the existence of such intermediate *in vivo*. Indeed, the concentrations of rPrP used to study the *in vitro* polymerization are far above those observed *in vivo* and buffers involved are not compatible with life. But, in the view presented here, micelles play the important role to sustain the conformation that is eligible for the amyloid formation. This suggests that the PrP^C^ should reach a specific conformation to be able to polymerize into amyloid. What happens *in vivo*? It was recently shown that:

1. the conformational structure and stability of the recombinant human PrP in a membrane environment are substantially different from those of the free protein in solution [18], [19]
2. anionic lipids bicelles converted α-helix-rich rPrP to a β-sheet conformation [15],
3. lipid is necessary to convert PrP^C^ to infectious PrP^Sc^ under physiological conditions [20], [21],
4. the hydrophobic highly conserved middle region of PrP is involved in the interaction with lipid membranes [22], and deletion in this part of the molecule impaired PrP^Sc^ induced conversion [23]. This phenomenon was also observed for other amyloid forming peptides [14], [24].

Thus our hypothesis is the formation of mixed-micelles containing phospholipids and rPrP reducing the concentration necessary to reach CMC (Critical Micellar Concentration) under physiological conditions. We believe that it is consistent with the appearance of nucleation, *in vivo*, at low concentration of proteins.

The main characteristic of the *in vivo* formed amyloids is infectiosity and this property is related to the amyloid structure [25]. It is important to remind that most of the amyloids produced *in vitro* are not infectious. However, recently it was shown that addition of phospholipids [21] during *in vitro* polymerization leads consistently to infectious amyloids [20], [26]. Furthermore, it was proven that rPrP proteins interact with membrane phospholipids [19] and this interaction precedes conformational changes [18], a phenomenon also observed for other amyloid forming peptides [14]. Our hypothesis of formation of mixed-micelles containing phospholipids and rPrP, in such mixed-micelles, as in pure prion micelles, allow the protein to reach the PrP* conformation competent to generate infectious amyloids.

## Materials and Methods

### General assumptions on the model

This model is used to fit data, that is why we have to give assumptions on rates to obtain a physical and biological relevant model. In the case of micelles(or 3D spherical structures as it is observed experimentally here), we assume as in [27] that assimilation rates 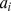 and 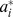 are constant for any 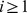 and we denote them by 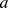 and 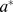 respectively, both of them being positive constants. Indeed, we assume that both types of proteins have the same affinity with respect to micelles of any size. The depletion rate needs to take the spherical structure of a micelle into account, which radius linked to the number of monomers that composes it. In [27], this term is given under the form 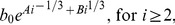
(18)


which is derived from chemical potential for one species of monomers. However, for the sake of simplicity we interpret differently this form and adapt it to one model. First, we suppose that in the smallest size, micelles do not transconformed PrP^C^ monomers into amyloid competent isoform. We justify this assumption by thermodynamic constraints, assumed to be stronger in the greatest size. As the term in 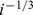 is dominant for small micelles, we let 
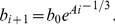
(19)


The term in 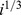 is dominant in the greatest size, thus this part is taken into account for the depletion of PrP*, 
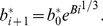
(20)


Now, for polymerization, we consider a constant polymerization rate 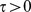, such that 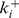 is equal to 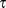 for any 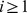, and a depolymerization rate 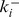 equal to a constant 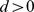 for 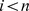, where 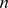 is the nucleus size and equal to 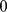 for longer polymer, 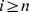, *i.e.* polymerization becomes an irreversible process after the nucleus is reached. Moreover, a linear splitting rate is taken, that is 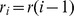 with 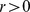 and a uniform kernel given by
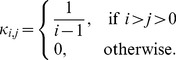
(21)


### Methods

#### Expression and purification of recombinant prion protein

**Recombinant** 90–231 prion protein (rPrP) was produced as described previously, see [10]. Protein concentrations were determined spectrophotometrically (Beckman spectrophometer) using an extinction coefficient of 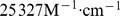 at 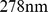 and a molecular mass of 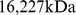. Purity of the protein preparation was assessed by phase reverse HPLC. The protein was stored lyophilized at 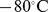.

#### Transmission electron microscopy

Samples were absorbed on carbon/formvar-coated copper grids (300 mesh) (Agar scientific, Saclay, France) and stained by negative contrast with 2% (w/v) uranyl acetate for one minute. Labelled samples are observed after negative contrast with uranyl acetate 2% on a JEOL 1200 EX II transmission electron microscope (Service commun de microscopie électronique de l’université Montpellier II, Montpellier, France) at 80KV of voltage. Number of micelles were counted directly on images and size of micelles as well as length and width of fibrils were measured using ImageJ software (http://rsbweb.nih.gov/ij/).

#### Kinetics measurements of polymerization

Kinetics of amyloid formation was monitored in SpectraMax Gemini XS (Molecular Devices). Samples containing 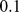 to 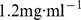 of the oxidized form of HaPrP90-231 (rPrP) were incubated in Buffer A, Buffer B or Buffer C (see [10] for more details on the buffers composition) upon continuous shaking at 1350 rpm in 96-well plates and in the presence of ThT (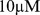). The kinetics was monitored by bottom-reading of fluorescence intensity using 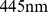 excitation and 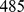 and 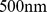 emission. Every set of measurements was performed in triplicates, and the results were averaged. Previously prepared amyloid were used to performed the seeding and the w/w per cent were calculated assuming that suspension was homogeneous.

#### Numerical simulation

A code was developed to simulate the equations and compared to data. It is available at http://www.ci2ma.udec.cl/ehingant/.

## Supporting Information

Figure S1 **Characterization by antibodies of micelles.** Transmission electron microscopy view of micelles occurring as spherical structures while PrP^Sc^ polymers appear as rigid-rod. The method used: 1. Samples absorption on carbon/formvar-coated copper grids (300 mesh) (Agar scientific, Saclay, France) 2. Labelling with antibodies: 1/antibody 3F4 (Covance, Berkeley, California; 1/100e in PBS 1% BSA), 2/antibody Rabbit anti Mouse (1/400e in PBS 1%BSA), 3/antibody Goat anti Rabbit-10 nm gold (1/100e in PBS BSA 1%) 3. Negative contrast staining with 2% (w/v) uranyl acetate for one minute.
(TIFF)

Figure S2
**Numerical simulation of the model in [14].** **a**, The normalized polymerization shape of the mass of polymers 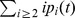. The simulation is done for a range of seeding (initial mass of PrP^Sc^) given by 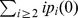 ranges from 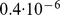 to 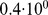 with a concentration of monomers PrP^C^ given by 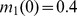 mg/ml. In this model there are no micelles, PrP^C^ monomers directly polymerize with PrP^Sc^. **b**, 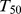 *vs.* seeding associated to the polymerization shape in (a), seeding is presented in log-scale. Both show the Lag time 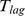 together with half time 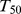 disappear when seeding increase.(TIFF)

Figure S3
**Dependency of slope to the initial concentration of PrP^C^.** Comparison of our model and the model in [14] with experimental results. The slope 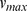 is the apparent polymerization rate at the inflexion point or with the model: the number 
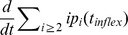
 where 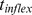 is the inflexion time such that 
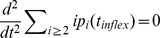
. **a**, Experimental data obtained in different buffers. The slope is obtained by fitting them with a sigmoidal shape. Red dot or the mean obtained through the experiments. **b**, Result obtained with the nucleation-dependent model [14] and **c**, with the micelle-dependent model presented in the paper. The micelle-dependent model, item (c), appears in a better agreement as a convex function.(TIFF)

Figure S4
**Dependency of the Half-time to a postpone seeding.** Circle is the mean with standard deviation of the half-time over 3 experiments. Meanwhile, the solid line is the result provided by the micelle-dependent model and the dashed line is the theoretical Half-time when pure nucleation-dependent polymerization is considered. This experiment consists in 4 test tubes containing 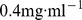 of PrP^C^ monomers in the same buffer at time 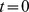. Then, at 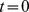 the first tube is seeded with preformed polymers this leads to a half-time set as the 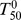. The second test tube is seeded 1 hour later (the seeding time) with the same concentration of polymers and so forth. The 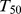 is supposed to be linearly brought forward (dashed line) according to the seeding time, in the case where nothing happens between the beginning of the experiments and the time when polymers are introduced. Experimentally this time is less than the one expected (circle). The formation of micelles and transconformed monomers at the early phase explains this situation. Indeed, the process had already started with the micelles formation and active monomers PrP* in the buffer, before seeding. Thus, polymerization starts earlier than expected.(TIFF)

Figure S5 **The half-time vs initial concentration of PrP^C^.** It appears that the decreasing rate in log scale of the half-time remains independent on the nucleus size *n* for the micelle-dependent model which is not consistent with the nucleation-dependent model.
(TIFF)

Figure S6 **Transmission electron microscopy's views of the experiments at different times.** The images represent an arbitrary selection among many ones selected from the beginning to the end of experiments in two different buffers corresponding to Fig. 2, top (Buffer C) and bottom (Buffer B).
(TIFF)
