# Supplementary figures and images for: A Micellar On-Pathway Intermediate Step Explains the Kinetics of Prion Amyloid Formation
Source: PLoS Comput Biol. 2014 Aug 7;10(8):e1003735. doi: 10.1371/journal.pcbi.1003735 (PMC4125056; doi:10.1371/journal.pcbi.1003735)

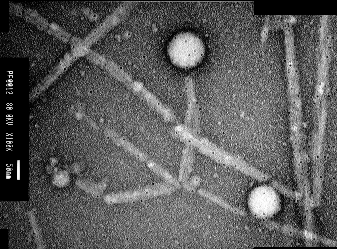

Supplement: Figure S1 — Characterization by antibodies of micelles. Transmission electron microscopy view of micelles occurring as spherical structures while PrPSc polymers appear as rigid-rod. The method used: 1. Samples absorption on carbon/formvar-coated copper grids (300 mesh) (Agar scientific, Saclay, France) 2. Labelling with antibodies: 1/antibody 3F4 (Covance, Berkeley, California; 1/100e in PBS 1% BSA), 2/antibody Rabbit anti Mouse (1/400e in PBS 1%BSA), 3/antibody Goat anti Rabbit-10 nm gold (1/100e in PBS BSA 1%) 3. Negative contrast staining with 2% (w/v) uranyl acetate for one minute. (TIFF) [file pcbi.1003735.s001.tiff]

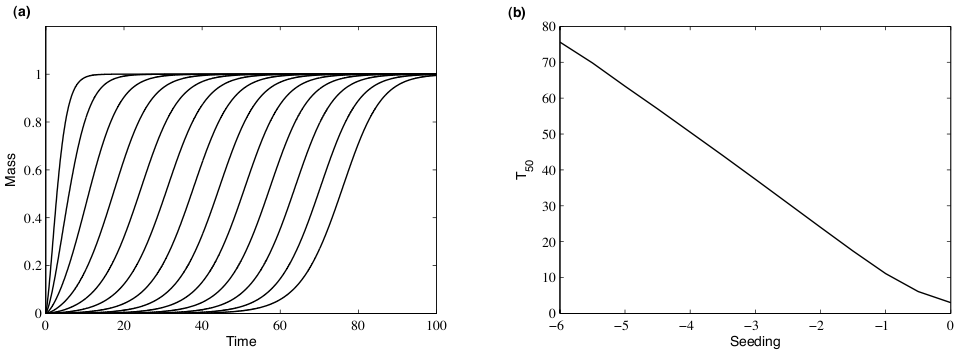

Supplement: Figure S2 — Numerical simulation of the model in [14]. a, The normalized polymerization shape of the mass of polymers . The simulation is done for a range of seeding (initial mass of PrPSc) given by ranges from to with a concentration of monomers PrPC given by mg/ml. In this model there are no micelles, PrPC monomers directly polymerize with PrPSc. b, vs. seeding associated to the polymerization shape in (a), seeding is presented in log-scale. Both show the Lag time together with half time disappear when seeding increase. (TIFF) [file pcbi.1003735.s002.tiff]

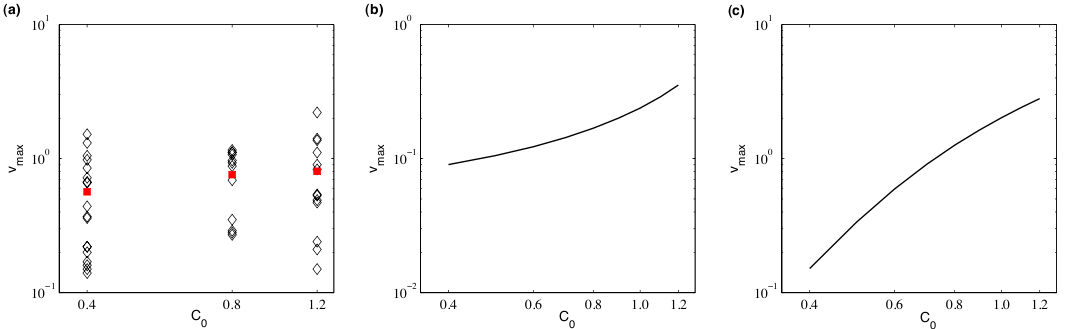

Supplement: Figure S3 — Dependency of slope to the initial concentration of PrPC. Comparison of our model and the model in [14] with experimental results. The slope is the apparent polymerization rate at the inflexion point or with the model: the number where is the inflexion time such that . a, Experimental data obtained in different buffers. The slope is obtained by fitting them with a sigmoidal shape. Red dot or the mean obtained through the experiments. b, Result obtained with the nucleation-dependent model [14] and c, with the micelle-dependent model presented in the paper. The micelle-dependent model, item (c), appears in a better agreement as a convex function. (TIFF) [file pcbi.1003735.s003.tiff]

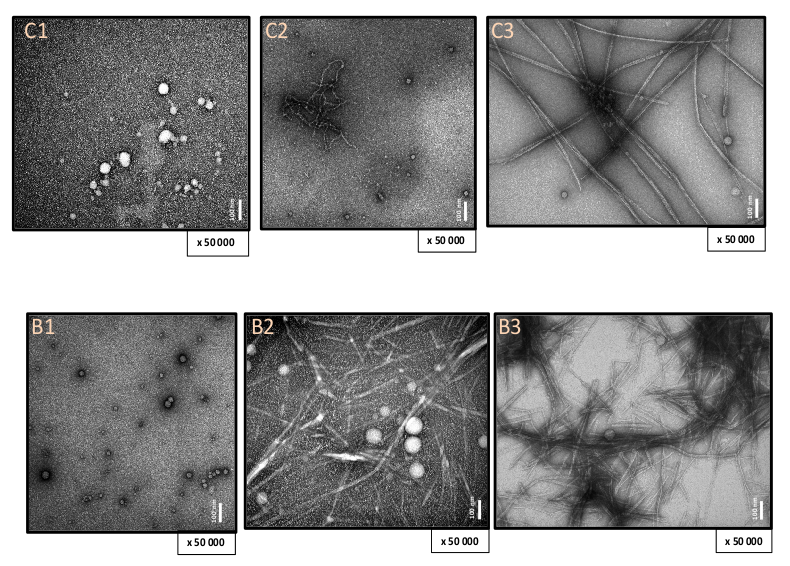

Supplement: Figure S6 — Transmission electron microscopy's views of the experiments at different times. The images represent an arbitrary selection among many ones selected from the beginning to the end of experiments in two different buffers corresponding to Fig. 2, top (Buffer C) and bottom (Buffer B). (TIFF) [file pcbi.1003735.s006.tiff]
